# Supplementary material for: Curcumin modulated gut microbiota and alleviated renal fibrosis in 5/6 nephrectomy-induced chronic kidney disease rats
Source: PLoS One. 2025 Jan 9;20(1):e0314029. doi: 10.1371/journal.pone.0314029 (PMC11717218; doi:10.1371/journal.pone.0314029)
Supplement: S1 Table — (DOCX) [file pone.0314029.s001.docx]

**Supplement Table 1 . Primer sets designed for quantitative real-time PCR.**

| **Gene** | **Primers** | **Sequence (5′ -3′ )** |
| --- | --- | --- |
| *GAPDH* | forward | GCCCAGAACATCATCCCTGCAT |
|  | reverse | GCCTGCTTCACCACCTTCTTGA |
| *Ocln* | forward | CACGTTCGACCAATGCTCTCTCA |
|  | reverse | ACTTCCTCCAAAGATGCCCGTTC |
| *Cldn1* | forward | CCAGGTATGAATTTGGCCAGGCT |
|  | reverse | AAGAGGTTGTTTTCCGGGGACAG |
| *ZO-1* | forward | AGCCTCAGAAACCTCAAGCGAAG |
|  | reverse | TCGGCTGACTCTGAGAATGGACT |
| *TNF-α* | forward | CAGCCTCTTCTCATTCCTGCTCG |
|  | reverse | CAGCCTCTTCTCATTCCTGCTCG |
| *IL-6* | forward | GCCACTGCCTTCCCTACTTCAC |
|  | reverse | CTGGTCTGTTGTGGGTGGTATCC |
| *IL-1β* | forward | AGTTTGAGTCTGCACAGTTCCCC |
|  | reverse | ACGGGTTCCATGGTGAAGTCAAC |
| *FN* | forward | GACTGTACTTGTCTGGGCGAAGG |
|  | reverse | CCCTCTGTTGTCCTTCTTGCTCC |
| *ACTA2* | forward | TGCTGTCCCTCTATGCTTCTGGA |
|  | reverse | CAGGGAAGAAGAGGAAGCAGCAG |
| *COLIA1* | forward | GCGAACAAGGTGACAGAGGCATA |
|  | reverse | AGCTGAAGTCATAACCACCGCTG |
| *MCP1* | forward | CTGCTGCTACTCATTCACTGGCA |
|  | reverse | TTGGGGTCAGCACAGATCTCTCT |
