# Supplementary material for: Curcumin modulated gut microbiota and alleviated renal fibrosis in 5/6 nephrectomy-induced chronic kidney disease rats
Source: PLoS One. 2025 Jan 9;20(1):e0314029. doi: 10.1371/journal.pone.0314029 (PMC11717218; doi:10.1371/journal.pone.0314029)
Supplement: S1 File — (DOC) [file pone.0314029.s003.doc]

**16S rDNA sequencing and gut microbiota analysis**

**DNA extraction and amplification**

Total genomic DNA was extracted using MagPure Soil DNA LQ Kit (Magan) following the manufacturer’s instructions. DNA concentration and integrity were measured with NanoDrop 2000 (Thermo Fisher Scientific, USA) and agarose gel electrophoresis. Extracted DNA was stored at -20°C until further processing. The extracted DNA was used as template for PCR amplification of bacterial 16S rRNA genes with the barcoded primers and Takara Ex Taq (Takara). For bacterial diversity analysis, V3-V4 (or V4-V5) variable regions of 16S rRNA genes was amplified with universal primers 343F (5’-TACGGRAGGCAGCAG-3’) and 798R (5’-AGGGTATCTAATCCT-3’) for V3-V4 regions .

**Library construction and sequencing**

The Amplicon quality was visualized using agarose gel electrophoresis. The PCR products purified with AMPure XP beads (Agencourt) and amplified for another round of PCR. After purified with the AMPure XP beads again, the final amplicon was quantified using Qubit dsDNA Assay Kit (Thermo Fisher Scientific,USA). The concentrations were then adjusted for sequencing. Sequencing was performed on an Illumina NovaSeq 6000 with 250 bp paired-end reads. (Illumina Inc., San Diego, CA; OE biotech Company; Shanghai, China).

**Gut microbiota analysis process**

**Bioinformatic analysis**

The library sequencing and data processing were conducted by OE biotech Co., Ltd. (Shanghai, China). Raw sequencing data were in FASTQ format. Paired-end reads were then preprocessed using Cutadapt software to detect and cut off the adapter. After trimming, paired-end reads were filtering low quality sequences, denoised , merged and detect and cut off the chimera reads using DADA2 with the default parameters of QIIME2 (2020.11). At last, the software output the representative reads and the ASV abundance table. The representative read of each ASV was selected using QIIME2 package. All representative reads were annotated and blasted against Silva database (Version 138) using q2-feature-classifier with the default parameters.

QIIME2 software was used for alpha and beta diversity analysis. The microbial diversity in samples was estimated using the alpha diversity that include Chao1 index,Shannon index,Simpson index and ACE index. The unweighted Unifrac distance matrix performed by R package was used for unweighted Unifrac Principal coordinates analysis (PCoA) to estimate the beta diversity. Then the R package was used to analyze the significant differences between different groups using ANOVA statistical test. The linear discriminant analysis effect size (LEfSe) method was used to compare the taxonomy abundance spectrum.

**LC-MS metabolomic analysis technology**

**Chemicals**

All chemicals and solvents were analytical or HPLC grade. Water, methanol, acetonitrile, formic acid were purchased from Thermo Fisher Scientific(Thermo Fisher Scientific, Waltham, MA, USA). L-2-chlorophenylalanine was from Shanghai Heng chuang Bio-technology Co., Ltd. (Shanghai, China).

**Sample Preparation**

**The pretreatment of serum sample**

80 μL of sample was added to a 1.5 mL Eppendorf tube with 10 μL of L-2-chlorophenylalanine (0.3 mg/mL) dissolved in methanol as internal standard, and the tube was vortexed for 10 s. Subsequently, 240 μL of ice-cold mixture of methanol and acetonitrile (2/1, vol/vol) was added, and the mixtures were vortexed for 1 min, and the whole samples were extracted by ultrasonic for 10 min in ice-water bath，stored at -20 ℃ for 30 min. The extract was centrifuged at at 4°C (13,000 rpm) for 10 min. 160 μL of supernatant in a glass vial was dried in a freeze concentration centrifugal dryer .240μL mixture of methanol and water (1/4, vol/vol) were added to each sample, samples vortexed for 30 s, extracted by ultrasonic for 3 min in ice-water bat, then placed at -20°C for 2 h. Samples were centrifuged at 4°C (13,000 rpm) for 10 min. The supernatants (150 μL) from each tube were collected using crystal syringes, filtered through 0.22 μm microfilters and transferred to LC vials.Finally, 15 μL supernatant was used for LC-MS detection.

**Data Preprocessing and Statistical Analysis**

The original LC-MS data were processed by software Progenesis QI V2.3 (Nonlinear, Dynamics, Newcastle, UK) for peak identification, retention time correction, peak alignment. Main parameters of 5 ppm precursor tolerance, 10 ppm product tolerance, and 5% product ion threshold were applied. Compound identification were based on precise mass-to-charge ratio (M/z), secondary fragments, and isotopic distribution using The Human Metabolome Database (HMDB), Lipidmaps (V2.3), Metlin, EMDB, PMDB, and self-built databases to do qualitative analysis. The extracted data were then further processed by removing any peaks with a missing value (ion intensity = 0) in more than 50% in groups, by replacing zero value by half of the minimum value, and by screening according to the qualitative results of the compound. Compounds with resulting scores below 36 (out of 60) points were also deemed to be inaccurate and removed. A data matrix was combined from the positive and negative ion data. Orthogonal Partial Least-Squares-Discriminant Analysis (OPLS-DA) were utilized to distinguish the metabolites that differ between groups.Variable Importance of Projection (VIP) values obtained from the OPLS-DA model were used to rank the overall contribution of each variable to group discrimination. A two-tailed Student’s T-test was further used to verify whether the metabolites of difference between groups were significant. Differential metabolites were selected with VIP values greater than 1.0 and p-values less than 0.05.
