# Supplementary material for: Curcumin modulated gut microbiota and alleviated renal fibrosis in 5/6 nephrectomy-induced chronic kidney disease rats
Source: PLoS One. 2025 Jan 9;20(1):e0314029. doi: 10.1371/journal.pone.0314029 (PMC11717218; doi:10.1371/journal.pone.0314029)

A chemiluminescence detection method was used to visualize bound antibodies on autoradiographic films.

Fig 2F

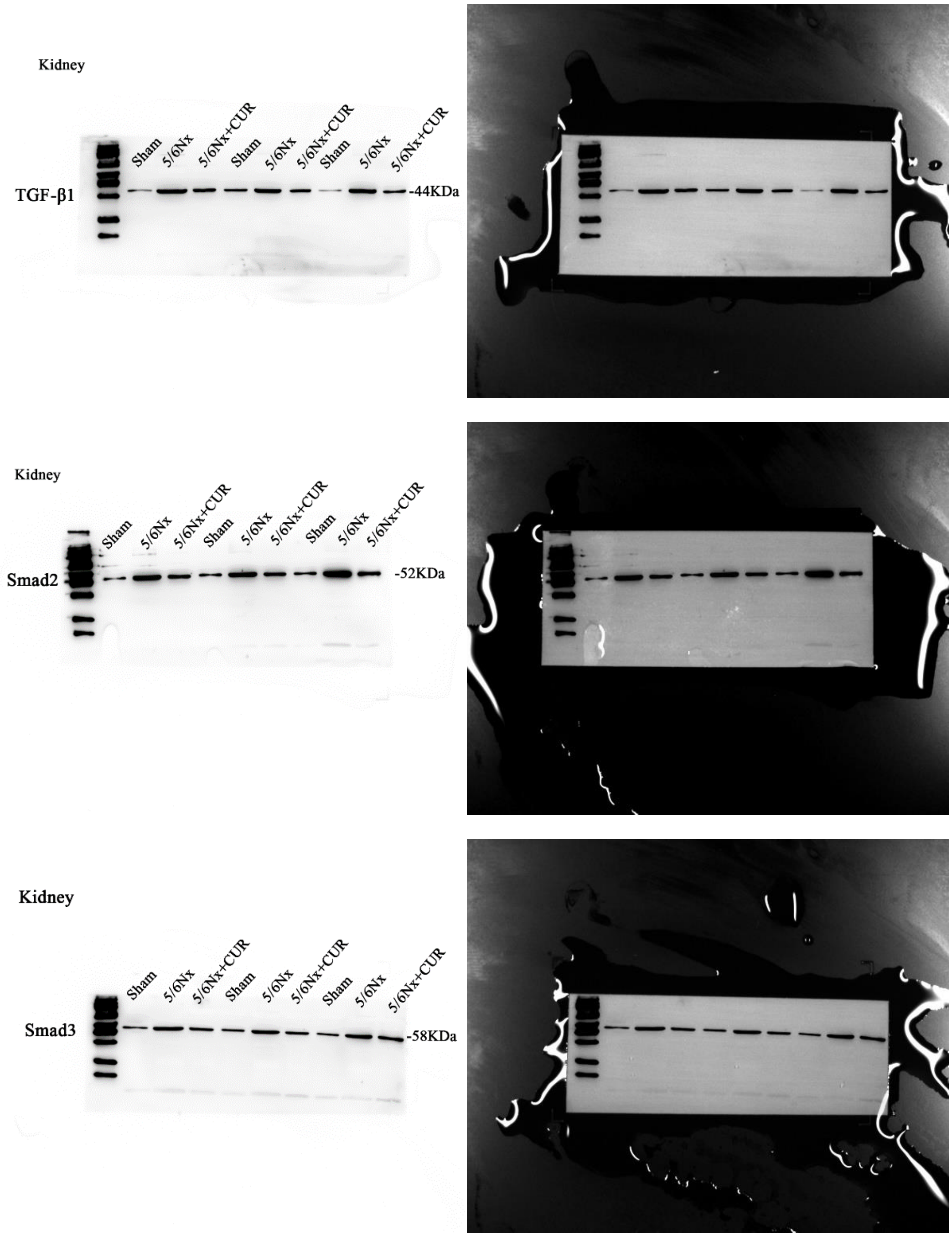

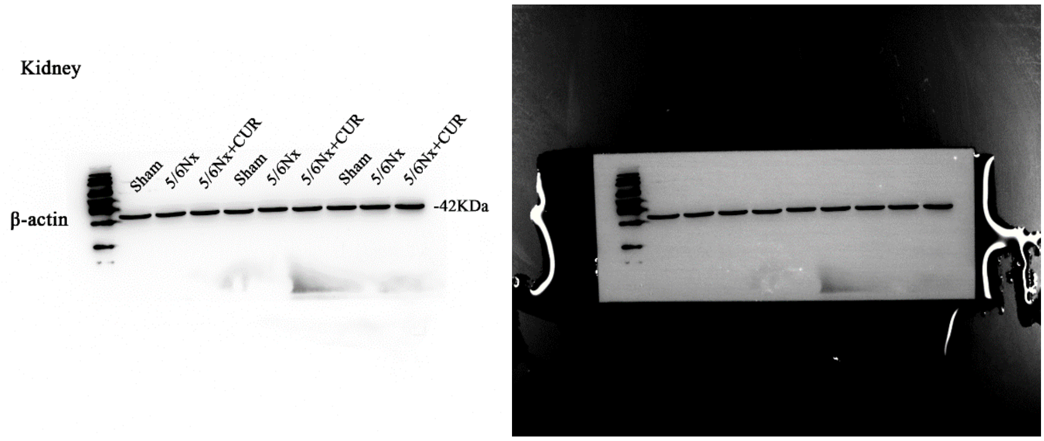
Kidney
Sham
5/6Nx
5/6Nx+CUR
Sham
5/6Nx
5/6Nx+CUR
Sham
5/6Nx
5/6Nx+CUR
β-actin
-42KDa

Fig 3E

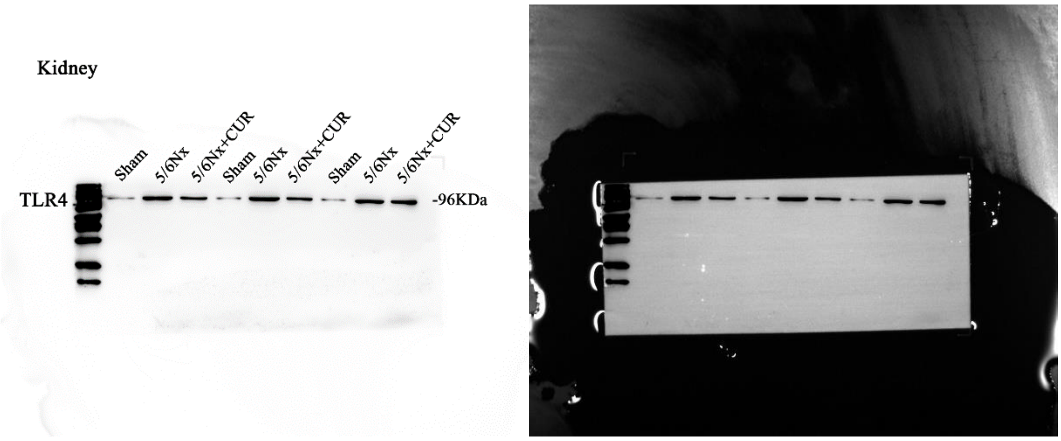
Kidney
Sham
5/6Nx
5/6Nx+CUR
Sham
5/6Nx
5/6Nx+CUR
Sham
5/6Nx
5/6Nx+CUR
TLR4
-96KDa

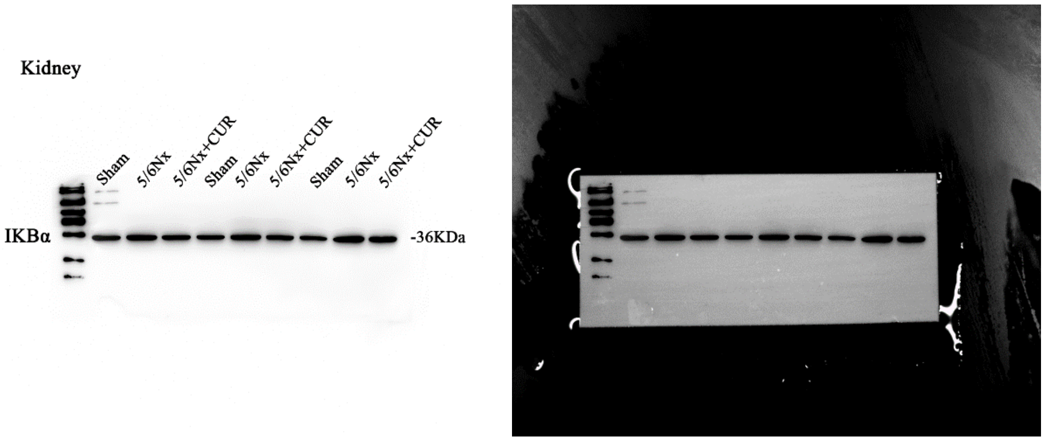
Kidney
Sham
5/6Nx
5/6Nx+CUR
Sham
5/6Nx
5/6Nx+CUR
Sham
5/6Nx
5/6Nx+CUR
IKBα
-36KDa

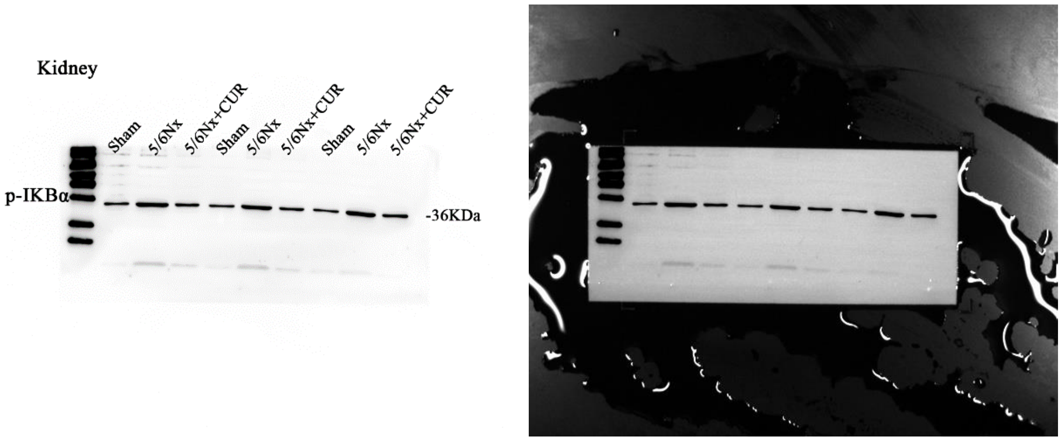

Kidney
Sham
5/6Nx
5/6Nx+CUR
Sham
5/6Nx
5/6Nx+CUR
Sham
5/6Nx
5/6Nx+CUR
p-IKBα
-36KDa

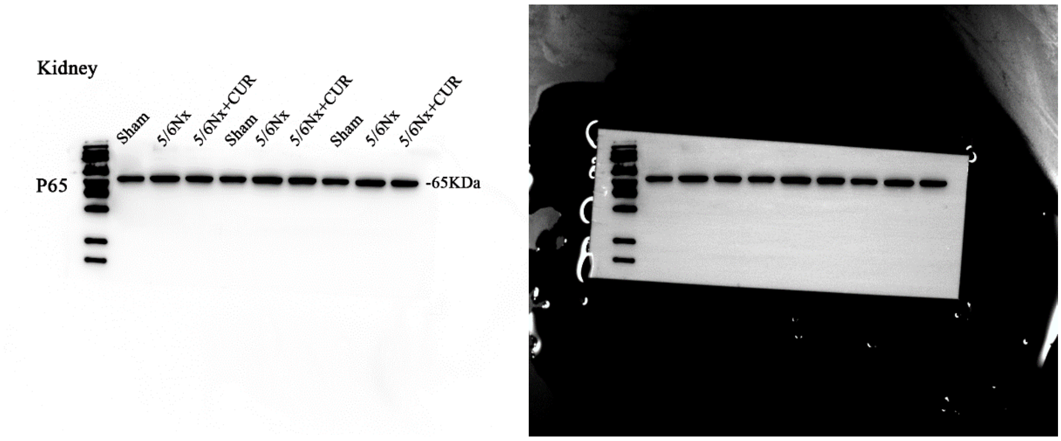

Kidney
Sham
5/6Nx
5/6Nx+CUR
Sham
5/6Nx
5/6Nx+CUR
Sham
5/6Nx
5/6Nx+CUR
P65
-65KDa

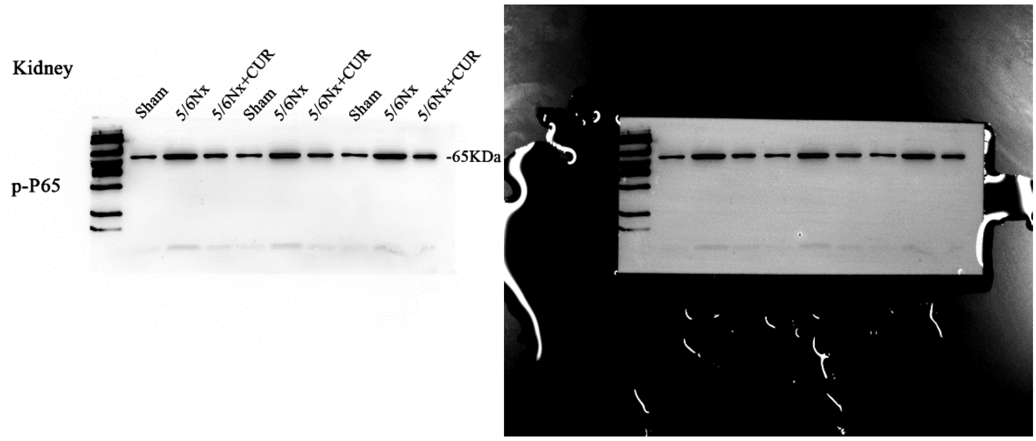

Kidney
Sham
5/6Nx
5/6Nx+CUR
Sham
5/6Nx
5/6Nx+CUR
Sham
5/6Nx
5/6Nx+CUR
p-P65
-65KDa

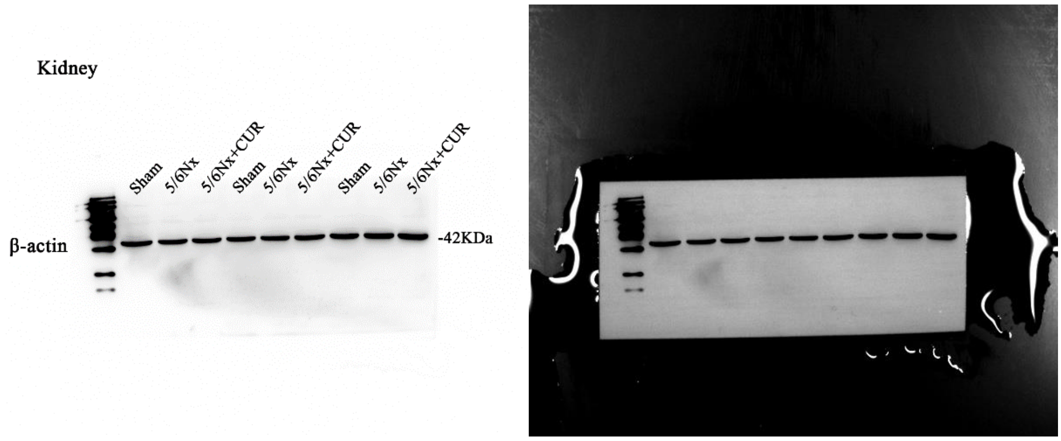

Kidney
Sham
5/6Nx
5/6Nx+CUR
Sham
5/6Nx
5/6Nx+CUR
Sham
5/6Nx
5/6Nx+CUR
β-actin
-42KDa

Fig 4I

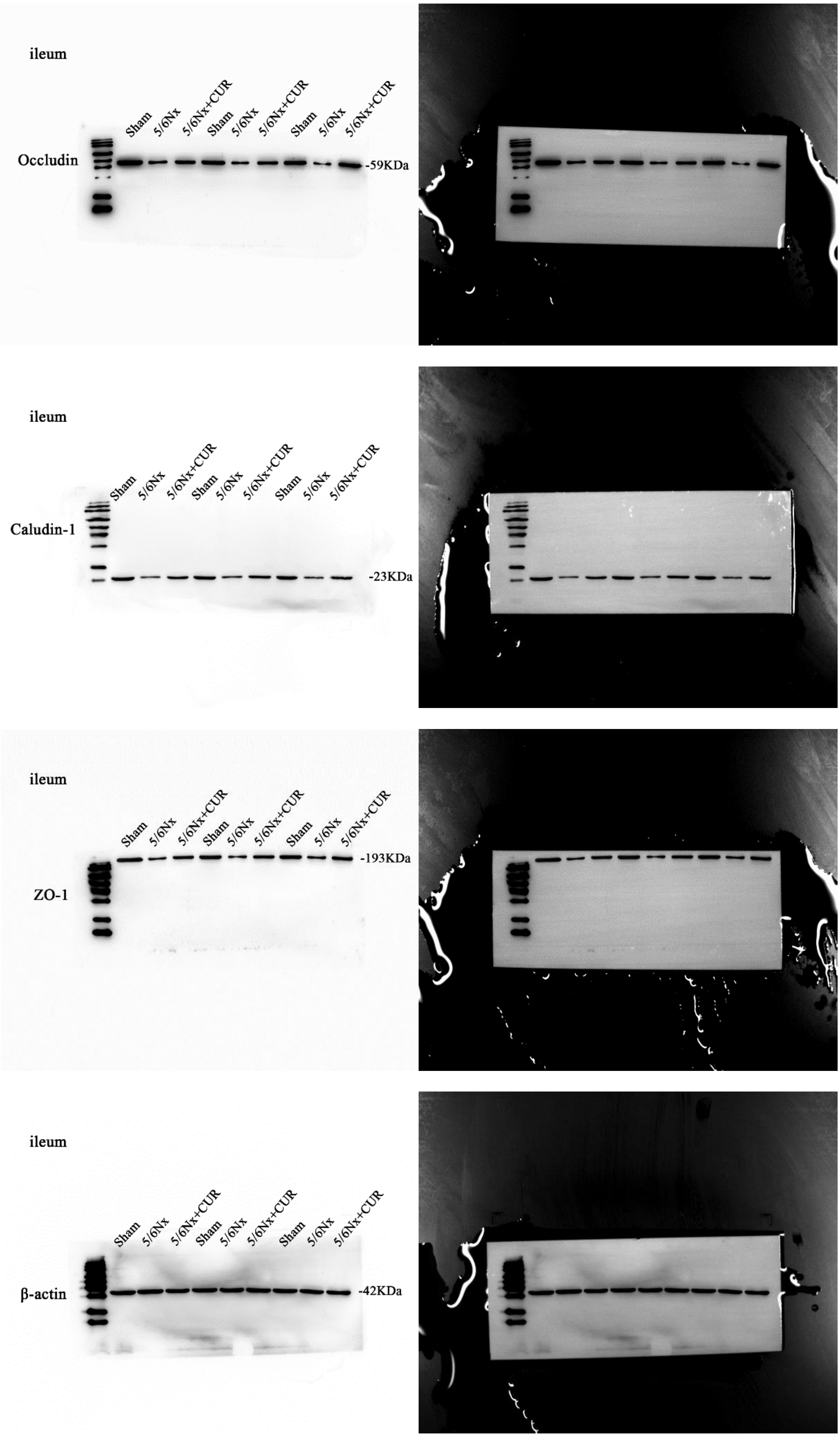
ileum
Sham
5/6Nx
5/6Nx+CUR
Sham
5/6Nx
5/6Nx+CUR
Sham
5/6Nx
5/6Nx+CUR
Occludin
-59KDa
ileum
Sham
5/6Nx
5/6Nx+CUR
Sham
5/6Nx
5/6Nx+CUR
Sham
5/6Nx
5/6Nx+CUR
Caludin-1
-23KDa
ileum
Sham
5/6Nx
5/6Nx+CUR
Sham
5/6Nx
5/6Nx+CUR
Sham
5/6Nx
5/6Nx+CUR
-193KDa
ZO-1
ileum
Sham
5/6Nx
5/6Nx+CUR
Sham
5/6Nx
5/6Nx+CUR
Sham
5/6Nx
5/6Nx+CUR
β-actin
-42KDa

Fig 4K

colon

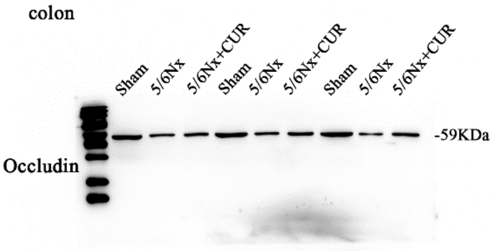

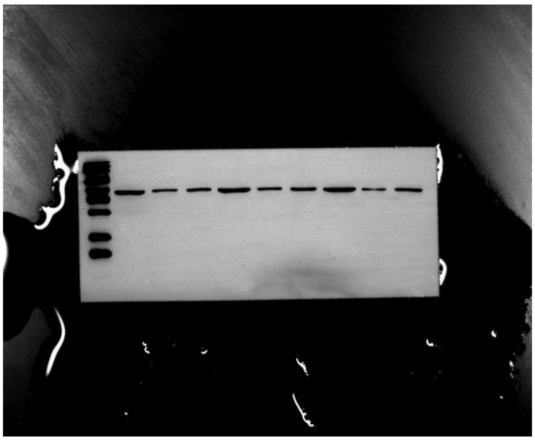

colon

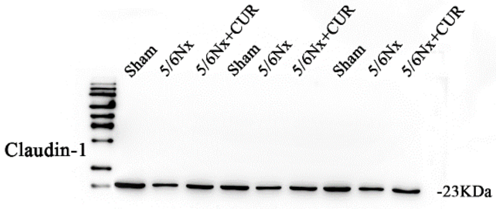

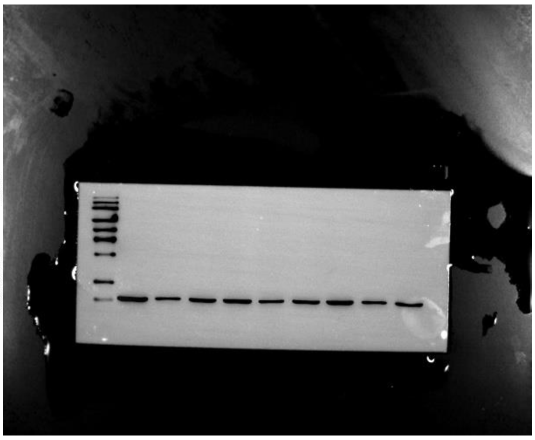

colon

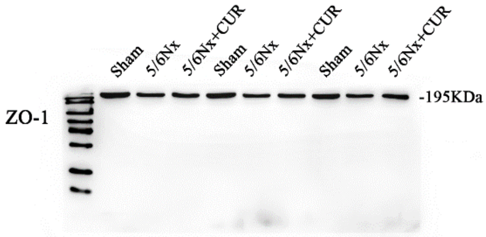

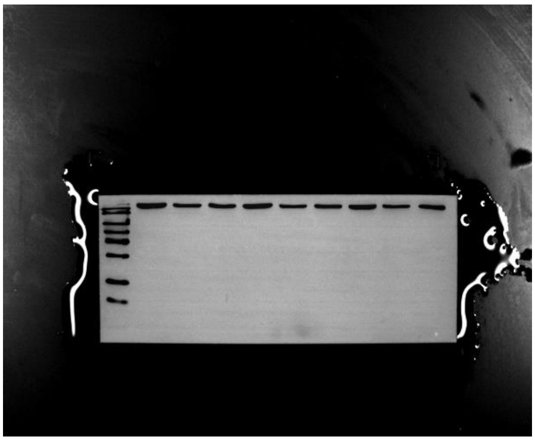

colon

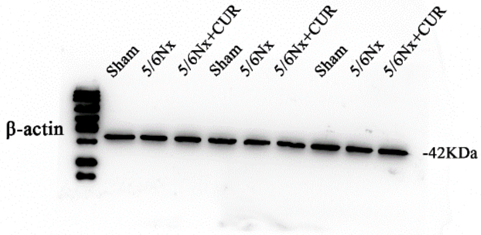

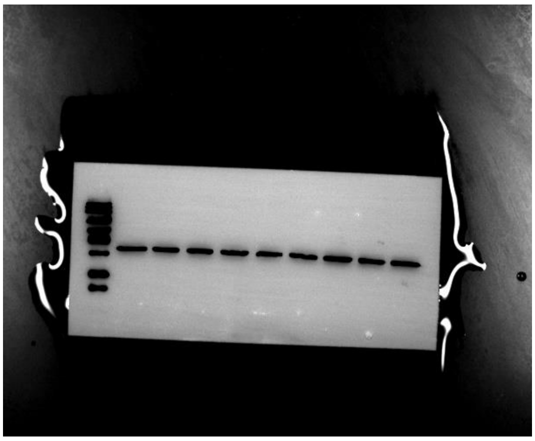

Supplement: S1 Raw images — (PDF) [file pone.0314029.s004.pdf]
