# Supplementary material for: Curcumin modulated gut microbiota and alleviated renal fibrosis in 5/6 nephrectomy-induced chronic kidney disease rats
Source: PLoS One. 2025 Jan 9;20(1):e0314029. doi: 10.1371/journal.pone.0314029 (PMC11717218; doi:10.1371/journal.pone.0314029)
Supplement: Attachment — Submitted filename: Response to Reviewers.doc [file pone.0314029.s005.doc]

Dear editor,

Thank you very much for your letter regarding our manuscript entitled “Curcumin modulated gut microbiota and alleviated renal fibrosis in 5/6 nephrectomy-induced chronic kidney disease rats”. We also thank the reviewers’ constructive comments and suggestions. Our point-by-point response to the reviewers’ and suggestions are listed below again.

We would like to submit the revised manuscript, and hope it is acceptable for publication in the journal.

Sincerely yours,

Cheng Li

Here are our responses to the reviewers’ comments one-by-one again.

**Journal Requirements:**

When submitting your revision, we need you to address these additional requirements.

1. Please ensure that your manuscript meets PLOS ONE's style requirements, including those for file naming. The PLOS ONE style templates can be found at https://journals.plos.org/plosone/s/file?id=wjVg/PLOSOne_formatting_sample_main_body.pdf and

<https://journals.plos.org/plosone/s/file?id=ba62/PLOSOne_formatting_sample_title_authors_affiliations.pdf>

**Response:** Thank you for your reminder.We have revised the manuscript according to the template you provided.

2. To comply with PLOS ONE submissions requirements, in your Methods section, please provide additional information regarding the experiments involving animals and ensure you have included details on (1) methods of sacrifice, (2) methods of anesthesia and/or analgesia, and (3) efforts to alleviate suffering.

**Response:** Thank you for your valuable suggestion.(1) methods of sacrifice:in our study, we utilized isoflurane as an inhalant anesthetic, and the rats were subsequently euthanized. (2) methods of anesthesia and/or analgesia：anesthesia was induced with 4% isoflurane prior to surgery and maintained with 2.5% isoflurane throughout the procedure. (3) efforts to alleviate suffering:all experimental protocols adhered to the 3R principle (reduction, replacement, and refinement) to minimize pain and distress, with animals handled by trained personnel in a calm manner. This information has been incorporated into the Methods section of our manuscript.

Thank you for your comments.

3. Thank you for stating the following financial disclosure:

"This project was assisted by National Natural Science Foundation of the People’s Republic of China (82270791), Jiangxi Traditional Chinese Medicine Science and Technology Plan Project (2022A137, 2023B1287), Jiangxi Provincial Health Commission Science and Technology Program (202212007), Science and Technology Research Project of Jiangxi Provincial Department of Education (GJJ201819, GJJ211805), Beijing Medical and Health Foundation (TYU046B) and Beijing Medical Award Foundation (YXJL-2022-0734-0294)."

 Please state what role the funders took in the study. If the funders had no role, please state: "The funders had no role in study design, data collection and analysis, decision to publish, or preparation of the manuscript."

If this statement is not correct you must amend it as needed.

Please include this amended Role of Funder statement in your cover letter; we will change the online submission form on your behalf.

**Response:** Thank you for your reminder. This work was supported by National Natural Science Foundation of the People’s Republic of China awarded to PuXun Tian (82270791) (https://kd.nsfc.cn/fundingProjectInit). Xulong Chen is supported by BELJING MEDICAL AWARD FOUNDATION (YXJL-2022-0734-0294) (https://www.yxjl.org/cms/html/index), BEUING MEDICAL AND HEALTH FOUNDATION (TYU046B) (http://www.ywjjh.org.cn/), Science Technology Research Project of Jiangxi Provincial Department of Education (GJJ211805), Jiangxi Traditional Chinese Medicine Science and Technology Plan Project (2023B1287) and Science and Technology Research Project of Jiangxi Provincial Department of Education (GJJ211805). Cheng Li received support from Jiangxi Traditional Chinese Medicine Science and Technology Plan Project (2022A137), Jiangxi Provincial Health Commission Science and Technology Program (202212007), Science and Technology Research Project of Jiangxi Provincial Department of Education (GJJ201819). The funders had no role in study design, data collection and analysis, decision to publish, and preparation of the manuscript.

Thank you for your comments.

4. When completing the data availability statement of the submission form, you indicated that you will make your data available on acceptance. We strongly recommend all authors decide on a data sharing plan before acceptance, as the process can be lengthy and hold up publication timelines. Please note that, though access restrictions are acceptable now, your entire data will need to be made freely accessible if your manuscript is accepted for publication. This policy applies to all data except where public deposition would breach compliance with the protocol approved by your research ethics board. If you are unable to adhere to our open data policy, please kindly revise your statement to explain your reasoning and we will seek the editor's input on an exemption. Please be assured that, once you have provided your new statement, the assessment of your exemption will not hold up the peer review process.

**Response:** Thank you for your reminder. All gut microbiome dataset files can be found online at <https://www.ncbi.nlm.nih.gov/bioproject/PRJNA1069977>

Additionally, four supplementary materials have been uploaded in the submission system. These four supplementary materials are as follows: **S1 Table**. Primer sets designed for quantitative real-time PCR. **S2 Table**. Differential metabolites between sham group and 5/6Nx group. **S1 File**. Bioinformatics Analysis of 16S rDNA Sequencing and LC-MS metabolomic analysis technology. **S1 Raw images**. The original uncropped and unadjusted images underlying all blot or gel data.

Thank you for your comments.

5. PLOS requires an ORCID iD for the corresponding author in Editorial Manager on papers submitted after December 6th, 2016. Please ensure that you have an ORCID iD and that it is validated in Editorial Manager. To do this, go to ‘Update my Information’ (in the upper left-hand corner of the main menu), and click on the Fetch/Validate link next to the ORCID field. This will take you to the ORCID site and allow you to create a new iD or authenticate a pre-existing iD in Editorial Manager. Please see the following video for instructions on linking an ORCID iD to your Editorial Manager account: https://www.youtube.com/watch?v=_xcclfuvtxQ

**Response:** Thank you for your suggestion. Our corresponding author has applied for a new ORCID iD (0000-0002-1408-9910).

Thank you for your comments.

**Reviewers Requirements:**

1- Why the authors did not add in vitro experiments to verify the in vivo results?

**Response:** Good question. The lack of *in vitro* experiments in this manuscript are mainly due to the following reasons:

1. Many *in vitro* studies^[1-4]^ have shown that curcumin inhibits epithelial-mesenchymal transition (EMT) of rat tubular epithelial cells (NRK) or human HK-2 cells stimulated by TGF-β1. Therefore, conducting *in vitro* experiments on curcumin is not the focus of our study.
2. Our study used fecal 16S rDNA gene sequencing to investigate the composition of gut microbiota in 5/6 nephrectomy rats. 16S sequencing is currently the sequencing method used in most articles on gut microbiota, but this sequencing method can only detect at the genus level and cannot be precise at the strain level. Therefore, it restricted our ability to extract strains for *in vitro* intervention on TGF-β1-induced NRK or human HK-2 cells. In subsequent experiment, we will use metagenomic sequencing to conduct in-depth exploration of bacterial strains.Although this study did not use metagenomic sequencing to screen strains for *in vitro* experiments, it still confirmed that curcumin reshape the composition of the gut microbiome as well as its metabolites in animal model of renal fibrosis, laying the foundation for future research in this direction.
3. Searching for articles published in journals of the same level in recent years, many articles related to gut microbiota do not have *in vitro* experimental data^[5-8]^.

Thank you for your comments.

2-More details are needed on how the authors selected the dose and treatment period for , Curcumin? Please supplement the relevant references.

**Response:** Thank you for your valuable question. The dose of curcumin selected for this study is 200 mg/kg/d, and the treatment period is 12 weeks.Related references for dose selection of curcumin^[9-13]^, relevant references for treatment period for curcumin^[14-16]^.

Thank you for your comments.

3- Why isn't there a separate curcumin group? Are additional effects of curcumin considered?

**Response:** Good question. This study did not set up a blank administration group, mainly considering the following factors:

1. The purpose of this study is to confirm that curcumin improves renal fibrosis in a 5/6Nx model; whether curcumin can reshape the intestinal flora and its metabolites in 5/6Nx rats and preliminary exploration of the relationship between gut bacteria and metabolites under the action of curcumin and renal fibrosis. Therefore, we sought to investigate the role of curcumin in a specific disease model, and no normal administration group was established.

2. As a safe natural metabolite, our study focuses on studying the role of curcumin in reshaping gut microbiota in 5/6 nephrectomy rats, rather than conducting toxicity experiments;

3. Referring to previous published literature, there are many articles on curcumin and gut microbiota in immune inflammatory diseases that do not add blank administration groups^[17-19]^.

Thank you for your comments.

4- It is recommended to briefly describe the CKD model building method, and attach relevant references as in the article.

### Response: Thank you for your question. I'm sorry for not providing a detailed description of the CKD model building method.CKD model construction is as follows:

### Anesthesia was induced with inhaled 4% isoflurane before surgery and maintained with 2.5% isoflurane during surgery. The back of rats were depilated and prepared for surgery. Anesthetized rats were fixed in a prone position on a mouse board, the skin preparation area was disinfected with 75% alcohol, and sterile surgical drapes (self-made gauze) were placed. A longitudinal 2 cm surgical incision was made next to the spine at the lower left costal margin, the skin and subcutaneous tissue were cut layer by layer and the muscle was bluntly separated. Next, gently lifted the perirenal fat sac with forceps, placed the surgical handle on it, exposed and fixed the kidney outside the incision, peeled off the renal capsule and perirenal fat, exposed the upper and lower poles of the kidney, used a sharp blade to remove the upper and lower poles of the kidney, and quickly compressed the renal section with gelatin sponge for 30 seconds to stop bleeding, then returned the kidney and sutured layer by layer. One week later, the right kidney was exposed using the same method. After peeling off the renal capsule and perirenal fat, the 4-0 silk thread was used to ligate the renal pedicle along the renal hilum. Then, the right kidney was removed and sutured layer by layer after confirming no bleeding. The sham operation was performed only to peel the renal membrane without damaging the kidney. The other steps are the same as above. After operation, the rats were fed and watered freely in an SPF environment at room temperature and 45% relative humidity^[20,21]^.

The above contents have been included in the methodology of the manuscript.Thank you for your comments.

5- It is recommended to supplement the dilution ratio of relevant antibody indicators, and supplement the experimental procedure basis of important experiments, such as the source of the kit used in the pcr experiment.

Response: We sincerely thank the reviewer for the careful reading of our manuscript. According to the reviewer's suggestion, we have added the dilution ratio of relevant antibody indexes in the “western blot analysis” of materials and methods. The source information of the kit used in the PCR experiment was supplemented in “RNA extraction and Real-Time PCR” (**S1 Table**).

Thank you for your comments.

6- It is recommended to increase the description of important details of 16S rDNA sequencing and non-targeted metabolome-related experiments, as current methods are too simplistic. The innovation of this paper lies in the application of multiple omics, but it is inappropriate for the author to put all the omics methods in the supporting materials.

**Response:** Thank you for your advice. We are sorry for not providing a detailed description of 16S sequencing and metabolomics methods in the method section. The description of important details of 16S rDNA sequencing and non-targeted metabolome-related experiments are as follows:

**16S rDNA gene sequencing**

Total genomic DNA was extracted using MagPure Soil DNA LQ Kit (Magan) following the manufacturer’s instructions. DNA concentration and integrity were measured with NanoDrop 2000 (Thermo Fisher Scientific, USA) and agarose gel electrophoresis. Extracted DNA was stored at -20°C until further processing. The extracted DNA was used as template for PCR amplification of bacterial 16S rRNA genes with the barcoded primers and Takara Ex Taq (Takara). For bacterial diversity analysis, V3-V4 (or V4-V5) variable regions of 16S rRNA genes was amplified with universal primers 343F (5’-TACGGRAGGCAGCAG-3’) and 798R (5’-AGGGTATCTAATCCT-3’) for V3-V4 regions. The Amplicon quality was visualized using agarose gel electrophoresis. The PCR products purified with AMPure XP beads (Agencourt) and amplified for another round of PCR. After purified with the AMPure XP beads again, the final amplicon was quantified using Qubit dsDNA Assay Kit (Thermo Fisher Scientific, USA). The concentrations were then adjusted for sequencing. Sequencing was performed on an Illumina NovaSeq 6000 with 250 bp paired-end reads. (Illumina Inc., San Diego, CA; OE biotech Company; Shanghai, China). Raw sequencing data were in FASTQ format. Paired-end reads were then preprocessed using cutadapt software to detect and cut off the adapter. After trimming, paired-end reads were filtering low quality sequences, denoised, merged and detected and cut off the chimera reads using DADA2 with the default parameters of QIIME2 (2020.11). At last, the software output the representative reads and the ASV abundance table. The representative read of each ASV was selected using QIIME2 package. All representative reads were annotated and blasted against Silva database Version 138 (or Unite) (16s/18s/ITS rDNA) using q2-feature-classifier with the default parameters.

**LC/MS untargeted metabolomics analysis**

80 μL of sample was added to a 1.5 mL Eppendorf tube with 10 μL of L-2-chlorophenylalanine (0.3 mg/mL) dissolved in methanol as internal standard, and the tube was vortexed for 10 s. Subsequently, 240 μL of ice-cold mixture of methanol and acetonitrile (2/1, vol/vol) was added, and the mixtures were vortexed for 1 min, and the whole samples were extracted by ultrasonic for 10 min in ice-water bath, stored at -20 ℃ for 30 min. The extract was centrifuged at at 4°C (13,000 rpm) for 10 min. 160 μL of supernatant in a glass vial was dried in a freeze concentration centrifugal dryer .240μL mixture of methanol and water (1/4, vol/vol) were added to each sample, samples vortexed for 30 s, extracted by ultrasonic for 3 min in ice-water bat, then placed at -20°C for 2 h. Samples were centrifuged at 4°C (13,000 rpm) for 10 min. The supernatants (150 μL) from each tube were collected using crystal syringes, filtered through 0.22 μm microfilters and transferred to LC vials. Finally, 15 μL supernatant was used for LC-MS detection.

The original LC-MS data were processed by software Progenesis QI V2.3 (Nonlinear, Dynamics, Newcastle, UK) for baseline filtering, peak identification, integral, retention time correction, peak alignment, and normalization. Main parameters of 5 ppm precursor tolerance, 10 ppm product tolerance, and 5% product ion threshold were applied. Compound identification was based on precise mass-to-charge ratio (M/z), secondary fragments, and isotopic distribution using The Human Metabolome Database (HMDB), Lipidmaps (V2.3), Metlin, EMDB, PMDB, and self-built databases to do qualitative analysis. The extracted data were then further processed by removing any peaks with a missing value (ion intensity = 0) in more than 50% in groups, by replacing zero value by half of the minimum value, and by screening according to the qualitative results of the compound. Compounds with resulting scores below 36 (out of 60) points were also deemed to be inaccurate and removed. A data matrix was combined from the positive and negative ion data. The matrix was imported in R to carry out Principal Component Analysis (PCA) to observe the overall distribution among the samples and the stability of the whole analysis process. Orthogonal Partial Least-Squares-Discriminant Analysis (OPLS-DA) and Partial Least-Squares-Discriminant Analysis (PLS-DA) were utilized to distinguish the metabolites that differ between groups. To prevent overfitting, 7-fold cross-validation and 200 Response Permutation Testing (RPT) were used to evaluate the quality of the model. Variable Importance of Projection (VIP) values obtained from the OPLS-DA model were used to rank the overall contribution of each variable to group discrimination. A two-tailed Student’s T-test was further used to verify whether the metabolites of difference between groups were significant. Differential metabolites were selected with VIP values greater than 1.0 and *p*-values less than 0.05.

The above contents have been included in the methodology of the manuscript. Thank you for your comments.

7- The Figures in the manuscript is not clear enough and very fuzzy, it is recommended to revise according to the requirements of the magazine submission.

Response: We are very sorry about this problem. We have checked the clarity of the figures in the manuscript and re-uploaded the figures. Submission system in the process of synthesis of the final manuscript may compress the pictures, which may reduce the clarity of the pictures. Please click on the figure to the top right of the manuscript and select 'Click here to access/download' to obtain a clear image, as shown in the following figure:

Thank you for your comments.

8- No arrows were used to mark specific pathological changes on all pathological pictures, Please add it.

Response: Thank you for your careful reading. We are very sorry about this problem. We have annotated the lesion site in all pathological images.

Thank you for your comments.

9- You can see many groups in the protein results picture, but the article method does not seem to describe them in detail, please check.

**Response:** Thank you for your excellent reminder. We apologize for our carelessness. We have refined the relevant methodology in the "Western Blot Analysis" of the materials and methods of the manuscript.

Thank you for your comments.

Other comments：

1-It is suggested that supplement the recent three years of research references.

**Response:** Thanks for the reviewer’s suggestion.We have supplemented the research references of the past 3 years in the manuscript.

Thank you for your comments.

2-It is recommended to verify the appropriate selection and use of statistical methods. It is suggested to improve the selection basis and standard of statistical methods.

**Response:** Thank you for your suggestion.

One way ANOVA is a commonly used statistical analysis method to compare whether there is a significant difference between the means of two or more independent groups. One way ANOVA assumes that the data in each group follows a normal distribution, so this method can only be used for analysis when the dependent variable is quantitative and follows a normal distribution. There are two reasons for using One way ANOVA in our manuscript: 1. Our study compared the significant difference between the mean values of the three independent groups; 2. The grouped data in this study belong to normal distribution, and the normal distribution of the data were analyzed by SPSS software, part of the results were shown in the following figure: Shapiro Wilk normality test *p*-value>0.05.

1) Normal distribution test of serum creatinine (Scr) value in each group

2) Normal distribution test of alpha-SMA specific PCR in each group

3) Normal distribution test of weight value in each group

Thank you for your comments.

3-The flora should be written in italics, and the whole text should be revised uniformly.

**Response:** Thank you very much. We apologize for the writing error. According to your comment, we have had corrected the mistakes.

Thank you for your comments.

4- The article language should be carefully polished by a professional company or a native English speaker.

**Response:** Thank you very much for your suggestion. According to your comment, we have had the manuscript polished and corrected the grammatical problems.

Thank you for your comments.

**Reviewer #2:** This manuscript eveals that curcumin has potential anti-renal fibrosis effects, and its mechanism may be related to its regulation of the composition of intestinal flora. This manuscript has good value for the application of CUR in anti-renal fibrosis. However, whether the anti-renal fibrosis effect of CUR depends on the regulatory effect on intestinal flora and the specific regulatory mechanism requires further experiments to verify. Since this study is closely related to metabolism, the author should provide the type of feedstuff, feeding status, body weight and survival rate of each group of rats in the animal experiment.

**Response:** Thank you very much for your valuable suggestions.

The feed type provided for the rats in each group of this study was a common feed formula, consisting of 73.5% corn, 20% wheat bran, 5% fish meal, 1% cereal flour, and 0.5% salt. Before the experiment, all rats were generally in good condition, with shiny hair, sensitive reactions, and normal diet. The rats in the three groups were kept in separate cages. After modeling and administration, the rats in the sham groups were well fed, with markedly increased weight, usual activities, good appetite, and glossy, smooth fur. In contrast, food intake, body weight, and gain in body weight were significantly lower in the 5/6Nx groups compared with the sham group, and rats exhibited dull and yellow fur. After 12 weeks of CUR administration, compared with the 5/6Nx group, the rats in administration group showed improvements in their mental state and appetite. Body weight changes of the three groups of rats during the experiment were shown in Table1.All animals in the sham group and the treatment group survived to the end of study, whereas two animals in the 5/6Nx group died.

The above contents have been included in our manuscript. Thank you for your comments.

**Reference**

[1] Li R, Wang Y, Liu Y, Chen Q, Fu W, Wang H, Cai H, Peng W, Zhang X. Curcumin inhibits transforming growth factor-β1-induced EMT via PPARγ pathway, not Smad pathway in renal tubular epithelial cells. PLoS One. 2013; 8(3): e58848.

[2] Zhou X, Zhang J, Xu C, Wang W. Curcumin ameliorates renal fibrosis by inhibiting local fibroblast proliferation and extracellular matrix deposition. J Pharmacol Sci. 2014; 126(4): 344-50.

[3] Hu JB, Li SJ, Kang XQ, Qi J, Wu JH, Wang XJ, Xu XL, Ying XY, Jiang SP, You J, Du YZ. CD44-targeted hyaluronic acid-curcumin prodrug protects renal tubular epithelial cell survival from oxidative stress damage. Carbohydr Polym. 2018 Aug 1; 193: 268-280.

[4] Noonin C, Thongboonkerd V. Curcumin prevents high glucose-induced stimulatory effects of renal cell secretome on fibroblast activation via mitigating intracellular free radicals and TGF-β secretion. Biomed Pharmacother. 2024 May; 174: 116536.

[5] He L, Zuo Q, Ma S, Zhang G, Wang Z, Zhang T, Zhai J, Guo Y. Canagliflozin attenuates kidney injury, gut-derived toxins, and gut microbiota imbalance in high-salt diet-fed Dahl salt-sensitive rats. Ren Fail. 2024 Dec; 46(1): 2300314.

[6] Alemasi A, Gu L, Zhou Y. Gut microbiota in the association between obesity and kidney function decline: a metagenomics-based study in a rat model. Ren Fail. 2024 Dec; 46(1): 2328320.

[7] Gao Y, Yang R, Guo L, Wang Y, Liu WJ, Ai S, Woon TH, Wang Z, Zhai Y, Wang Z, Peng L. Qing-Re-Xiao-Zheng Formula Modulates Gut Microbiota and Inhibits Inflammation in Mice With Diabetic Kidney Disease. Front Med (Lausanne). 2021 Sep 16; 8: 719950.

[8] Yang N, Lan T, Han Y, Zhao H, Wang C, Xu Z, Chen Z, Tao M, Li H, Song Y, Ma X. Tributyrin alleviates gut microbiota dysbiosis to repair intestinal damage in antibiotic-treated mice. PLoS One. 2023 Jul 31;18(7): e0289364.

[9] Feng W, Wang H, Zhang P, Gao C, Tao J, Ge Z, Zhu D, Bi Y. Modulation of gut microbiota contributes to curcumin-mediated attenuation of hepatic steatosis in rats. Biochim Biophys Acta Gen Subj. 2017 Jul; 1861(7): 1801-1812.

[10] Zhao YH, Shen CF, Wang GJ, Kang Y, Song YH, Liu JW. Curcumin alleviates acute kidney injury in a dry-heat environment by reducing oxidative stress and inflammation in a rat model. J Biochem Mol Toxicol. 2021 Jan; 35(1): e22630.

[11] Ali BH, Abdelrahman A, Al Suleimani Y, Manoj P, Ali H, Nemmar A, Al Za'abi M. Effect of concomitant treatment of curcumin and melatonin on cisplatin-induced nephrotoxicity in rats. Biomed Pharmacother. 2020 Nov; 131: 110761.

[12] Liu Z, Shi B, Wang Y, Xu Q, Gao H, Ma J, Jiang X, Yu W. Curcumin alleviates aristolochic acid nephropathy based on SIRT1/Nrf2/HO-1 signaling pathway. Toxicology. 2022 Sep; 479: 153297.

[13] Li T, Jin J, Pu F, Bai Y, Chen Y, Li Y, Wang X. Cardioprotective effects of curcumin against myocardial I/R injury: A systematic review and meta-analysis of preclinical and clinical studies. Front Pharmacol. 2023 Mar 9; 14: 1111459.

[14] Pivari F, Mingione A, Piazzini G, Ceccarani C, Ottaviano E, Brasacchio C, Dei Cas M, Vischi M, Cozzolino MG, Fogagnolo P, Riva A, Petrangolini G, Barrea L, Di Renzo L, Borghi E, Signorelli P, Paroni R, Soldati L. Curcumin Supplementation (Meriva^®^) Modulates Inflammation, Lipid Peroxidation and Gut Microbiota Composition in Chronic Kidney Disease. Nutrients. 2022 Jan 5; 14(1): 231.

[15] Wang D, Yang Y, Zou X, Zheng Z, Zhang J. Curcumin ameliorates CKD-induced mitochondrial dysfunction and oxidative stress through inhibiting GSK-3β activity. J Nutr Biochem. 2020 Sep; 83: 108404.

[16] ALTamimi JZ, AlFaris NA, Al-Farga AM, Alshammari GM, BinMowyna MN, Yahya MA. Curcumin reverses diabetic nephropathy in streptozotocin-induced diabetes in rats by inhibition of PKCβ/p^66^Shc axis and activation of FOXO-3a. J Nutr Biochem. 2021 Jan; 87: 108515.

[17] Cui C, Han Y, Li H, Yu H, Zhang B, Li G. Curcumin-driven reprogramming of the gut microbiota and metabolome ameliorates motor deficits and neuroinflammation in a mouse model of Parkinson's disease. Front Cell Infect Microbiol. 2022 Aug 10; 12: 887407.

[18] Tu X, Ren H, Bu S. Therapeutic effects of curcumin on constipation-predominant irritable bowel syndrome is associated with modulating gut microbiota and neurotransmitters. Front Microbiol. 2023 Dec 14; 14: 1274559.

[19] Li S, You J, Wang Z, Liu Y, Wang B, Du M, Zou T. Curcumin alleviates high-fat diet-induced hepatic steatosis and obesity in association with modulation of gut microbiota in mice. Food Res Int. 2021 May; 143: 110270.

[20] Wang J, Wang Y, Liu Y, Cai X, Huang X, Fu W, Wang L, Qiu L, Li J, Sun L. Ferroptosis, a new target for treatment of renal injury and fibrosis in a 5/6 nephrectomy-induced CKD rat model. Cell Death Discov. 2022 Mar 22; 8(1): 127.

[21] Almeida PP, Thomasi BBM, Menezes ÁC, Da Cruz BO, Costa NDS, Brito ML, Pereira AD, Castañon CR, Degani VAN, Magliano DC, Knauf C, Tavares-Gomes AL, Stockler-Pinto MB. 5/6 nephrectomy affects enteric glial cells and promotes impaired antioxidant defense in the colonic neuromuscular layer. Life Sci. 2022 Jun 1; 298: 120494.

**Explanation:**

1）Considering that Professor Puxun Tian is the leader of our research group and also the main contributor to the funding of this manuscript. After discussion and unanimous decision by all authors, Professor Puxun Tian is the sole corresponding author, and Xulong Chen is adjusted to be the second author. All authors' statements of agreement and handwritten signatures are uploaded in the submission system as documents.
2）Any modifications in the manuscript are marked with a yellow background. In addition, Figures 2, 3, and 4 have been modified according to the standard format, and we have uploaded the modified clear images.
